# Genotype by environment interaction for growth traits of families of *Acacia melanoxylon* based on BLUP and GGE biplot

**DOI:** 10.3389/fpls.2025.1656136

**Published:** 2025-11-14

**Authors:** Shao-Ning Ruan, Liu-Ben Chen, Bo Zhang, Wei-Hao Zhang, Guo-Chang Ding, Guang-Qiu Cao, Long-Tai Ye, Shi-Bin He, Dong-Yang Shen, Qiang Yan

**Affiliations:** 1 College of Forestry, Fujian Agriculture and Forestry University, Fuzhou, Fujian, China; 2 Fujian Forestry Survey and Design Institute, Fuzhou, Fujian, China; 3 College of Landscape Architecture and Art, Fujian Agriculture and Forestry University, Fuzhou, Fujian, China; 4 Zhangpu Zhongxi State-owned Forest Farm in Fujian Province, Zhangzhou, Fujian, China; 5 Nan’an Luoshan State-owned Forest Farm in Fujian Province, Nan’an, Fujian, China; 6 Xiqin Teaching Forest Farm, Fujian Agriculture and Forestry University, Nanping, Fujian, China

**Keywords:** *Acacia melanoxylon*, best linear unbiased prediction, GGE biplot, regional trial, family selection

## Abstract

*Acacia melanoxylon* is a high-quality timber species renowned for its superior heartwood and wide global use. To assess its stability, adaptability, and productivity in Fujian Province, China, a regional trial involving 47 *A. melanoxylon* families was conducted across four sites. The study utilized best linear unbiased prediction (BLUP) breeding values were estimated using 5-year-old diameter at breast height (DBH) and tree height data, and genotype plus genotype-by-environment (GGE) biplots were used to evaluate families and trial sites. The biplot generated based on BLUP values for DBH revealed that the four trial sites clustered into two groups. Group 1 comprised sites ZX (Zhangpu Zhongxi State-owned Forest Farm, Zhangpu County), LX (Quanzhou Luoxi State-owned Forest Farm, Quanzhou City), and XQ (Nanping Xiqin Teaching Forest Farm, Nanping City), while Group 2 included site LS (Nan’an Luoshan State-owned Forest Farm, Nan’an City). Sites LX and XQ showed strong positive correlations with each other and moderate correlations with ZX, indicating similar family rankings. In contrast, LX was negatively correlated with LS, reflecting opposing trends in genotype performance. Site ZX exhibited high discriminative power and representativeness, identifying it as the most effective location for evaluating families. Families 38, 35, 9, 17, and 24 demonstrated a combination of high yield and stability, underscoring their potential for extensive cultivation. The measurement of DBH is more cost-effective and accurate compared to tree height, and it aligns with our breeding objectives. Therefore, this study primarily focused on DBH for selection, and the results provide a theoretical foundation for promoting *Acacia melanoxylon* in Fujian Province.

## 1 Introduction

*Acacia melanoxylon*, an evergreen tree native to Australia, is a multipurpose timber species prized for its rapid growth, nitrogen-fixing ability, and high-quality wood (Hussain et al., 2020; Martins et al., 2020; Pereira and Ferreira, 2021). Since China's systematic introduction of *Acacia* species in 1985, *A. melanoxylon* has become an important component of plantations in southern Fujian Province, serving both ecological restoration and timber production needs (Fan et al., 2016). While the cultivation of *A. melanoxylon* in China has expanded, its genetic improvement program faces a significant challenge: a lack of attention of genotype-by-environment (G×E) interactions. Previous studies focused on selecting superior families have been confined to single-site trials. The absence of regional multi-environment trials (METs) makes it impossible to determine whether a superior genotype in one location will perform well in others, severely limiting the efficiency and effectiveness of breeding and deployment strategies.

Regionalized trials are essential in cultivar breeding and promotion. By analyzing the performance of various genotypes across diverse environmental conditions, METs elucidate genotype-by-environment (GE) interactions (G×E). They identify high-yielding, stable, and adaptable varieties, determining optimal planting zones (Li et al., 2023; Prášil et al., 2025). Statistical models for MET data have progressed from early multi-site joint ANOVA to combinations of ANOVA with regression or principal component analysis (PCA). More recently, the widely used Additive Main Effects and Multiplicative Interaction (AMMI) model and genotype plus GE (GGE) biplot have become prominent (Istanbuli et al., 2025; Vymyslický et al., 2025; Yousaf et al., 2025). Breeders consistently emphasize integrating G and GE effects to select superior genotypes in field trials (He et al., 2025; Skrøppa and Fundova, 2025). Yan et al (Yan et al., 2007). compared the AMMI model and the GGE biplot, concluding that the GGE biplot outperforms the AMMI1 model in mega-environment analysis and genotype evaluation. The GGE biplot excels in combining discriminative power with representativeness in environmental assessment, a feature the AMMI analysis lacks. Currently, mixed linear models with random effects are extensively used in trial data analysis (Khan et al., 2024). Best linear unbiased prediction (BLUP) values, more reliable than observed values and suitable for unbalanced data, offer higher prediction accuracy (Yuan et al., 2022; Amrate et al., 2024).

In recent years, the integrated BLUP-GGE approach has gained growing recognition in forest tree breeding owing to its excellent performance in evaluating genotype-by-environment interactions. For example, this approach has been successfully employed to assess growth stability and select superior genotypes in a variety of tree species, including *Populus euramericana* (Liu et al., 2021), *Castanopsis hystrix* (Liu et al., 2023), and *Larix gmelinii* var. *principis-rupprechtii* (Mayr.) Pilger (Zheng et al., 2023). These studies have demonstrated the scientific validity of the method. However, despite its demonstrated effectiveness in other species, the integrated BLUP-GGE biplot approach has not yet been systematically applied to the genetic evaluation and selection of *Acacia melanoxylon* families in Fujian Province. To bridge this critical gap, the present study was conducted with the following objectives: (1) to comprehensively evaluate the growth performance and stability of 47 *A. melanoxylon* families across four representative test sites in Fujian using the integrated BLUP-GGE biplot methodology; (2) to delineate mega-environments and assess the discriminative power and representativeness of test sites; and (3) to identify superior families with specific or broad adaptation for targeted breeding programs.

## 2 Materials and methods

### 2.1 Test site overview

The study selected four trial sites located in the primary plantation regions of Fujian Province, China: Zhangpu Zhongxi State-owned Forest Farm (ZX), Quanzhou Luoxi State-owned Forest Farm (LX), Nan’an Luoshan State-owned Forest Farm (LS), and Nanping Xiqin Teaching Forest Farm (XQ). These sites were chosen to represent the diverse ecological conditions, including variations in topography and microclimate, typical for *Acacia melanoxylon* cultivation in southern China. Detailed site conditions, including geographic coordinates, elevation range, soil type, and key climate parameters, are provided in **Table 1**.

**Table 1:** Site conditions of each experimental site.

Site	Longitude	Latitude	Elevation/m	Average annual temperature/°C	Annual average rainfall/mm	Soil type	Annual frost-free period/d
ZX	117°34′E	24°17′N	140−250	20.2	1448	Mountain red soil	Basically frost-free
LX	118°33′E	24°17′N	200−350	19.5	1350−1600	Mountain red soil	285
LS	118°16′E	24°50′N	415−481	20	1500−1800	Sandy loam and coarse-grained red soil	Basically frost-free
XQ	118°12′E	26°56′N	200−450	17.3	1663	Red soil	268

### 2.2 Test materials and design

The regional trial involved 47 *Acacia melanoxylon* families. Families 1 through 17 were derived from seed material collected in 2018 from superior individual trees in Australia (**Table 2**). Families 18 to 47 originated from a primary clonal seed orchard of *A. melanoxylon* at Luoxi State-owned Forest Farm, located in Quanzhou City, Fujian Province. This orchard was established by grafting preselected elite trees. Seeds were collected in 2018, germinated at the Zhangpu Zhongxi Forest Farm nursery in the same year, and planted in 2019 across four trial sites in Fujian Province.

**Table 2:** Introduction families’ data from Australia.

Provenance	Family	State	Original places of materials collected	Longitude	Latitude	Elevation/m
14176	1	QLD10	ATHERTON	145°26′E	17°17′S	1022
16513	2	VIC10	NNW WELSHPOOL	146°22′E	38°34′S	250
16526	3	SA9	SE MOUNT GAMBIER	140°56′E	37°57′S	40
17075	4	VIC10	DIGGERS REST	144°44′E	37°37′S	180
18980	5	VIC10	GELLIBRAND RIVER	143°15′E	38°43′S	50
19002	6	NSW9	WILD CATTLE CK SF	152°49′E	30°13′S	600
19293	7	NSW25	TALBINGO	148°18′E	35°31′S	400
19322	8	VIC50	CRAWFORD RIVER	141°30′E	37°56S	427
19495	9	TAS0	BEULAH	146°22′E	41°27′S	300
19497	10	TAS15	SOUTH OF BURNIE	145°56′E	41°09′S	200
19505	11	TAS14	CYGNET DISTRICT	146°51′E	43°09′S	160
19509	12	TAS10	ROGER RIVER WEST	146°45′E	41°01′S	100
19782	13	VIC15	HIGHLANDS	145°25′E	37°05′S	650
19783	14	VIC9	WODONGA	146°54′E	36°12′S	250
20623	15	NSW5	MT CANOBALAS	149°01′E	33°20′S	1125
20667	16	NSW15	KANGAROO VALLEY	150°36′E	34°38′S	400
20809	17	VIC3	POOLAIJELO	141°07′E	37°15′S	120

The experimental forest uniformly adopts a random complete block design (RCB), setting up 3, 4, 7, and 7 replicates (blocks) respectively in XQ, LS, ZX and LX. Each plot consisted of three trees arranged in a single column (1 row × 3 trees) with spacing of 2 × 3 m. Annual surveys were conducted in the experimental plantation. Diameter at breast height (DBH) was measured at 1.3 meters above ground level using a diameter tape, with an accuracy of 0.1 cm. Tree height was measured using an ultrasonic hypsometer, precise to 0.1 m. The growth data for DBH and tree height from 5-year-old *Acacia melanoxylon* used in this study consisted of individual tree measurements.

### 2.3 Statistical analysis method

The statistical analysis followed an integrated BLUP-GGE biplot framework to ensure accurate genetic parameter estimation and intuitive visualization. The process began with the estimation of breeding values using a mixed linear model fitted with the lmer() function from the R lme4 package (Gao et al., 2025). The model was specified as follows:


$$X_{ijkl}=\mu +L_{i}+B_{j}\left(L_{i}\right)+F_{k}+L_{i}\times F_{k}+e_{ijkl}$$

Where *X_ijkl_* represents the observed DBH or tree height of the *k*-th family in the *j*-th block at the *i*-th location (Yuan et al., 2022), *μ* is the overall mean, *L_i_* is the fixed effect of location, *B_j_(L_i_)* is the random block effect nested within location, *F_k_* is the random family effect, *L_i_×F_k_* is the random family-by-location interaction effect, and *e_ijkl_* is the residual error.

The model was fitted using the Restricted Maximum Likelihood (REML) algorithm, and the Best Linear Unbiased Predictions (BLUPs) for each family were extracted as the estimated breeding values. Prior to further analysis, the model’s adequacy was rigorously validated; the normality of residuals was assessed using a Q-Q plot, and the assumption of homoscedasticity was verified by plotting residuals against fitted values, with no substantial violations observed. In addition, significance testing for the fixed and random effects in the model was performed using the anova() and ranova() functions from the lmerTest package.

Finally, the validated BLUP values were used as inputs to generate GGE biplots using the GGEBiplotGUI package. The following parameter settings were selected based on the study’s objective to effectively visualize the genetic (G) and genotype-by-environment (GE) interaction patterns: Scaled = 0 (no standardization), Centering = ‘G+GE’ (focused on the GGE model), and singular value partitioning (SVP) = ‘JK’ (symmetric scaling focused on genotype discrimination) (Li et al., 2022).

## 3 Results

### 3.1 Estimation of breeding values using the BLUP method

**Figure 1:**
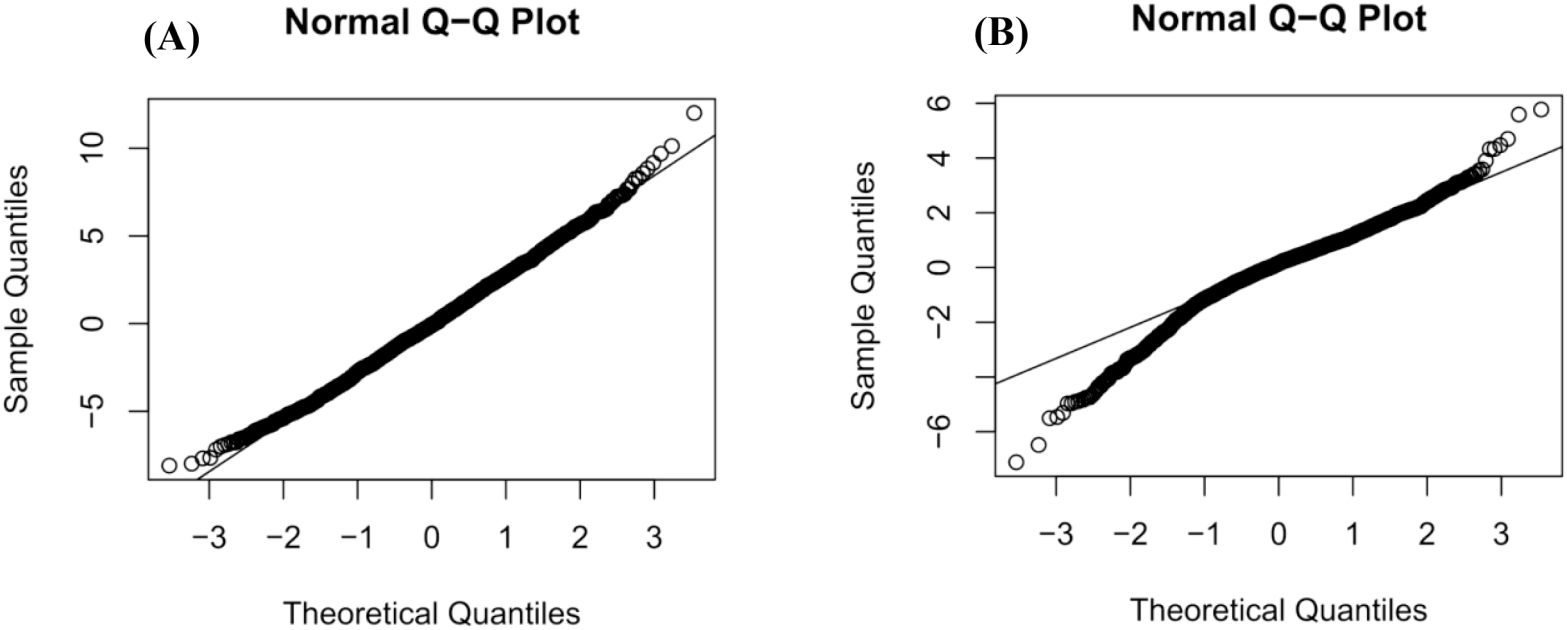
Normality test of mixed model residuals: **(A)** is DBH; **(B)** is tree height.

**Figure 2:**
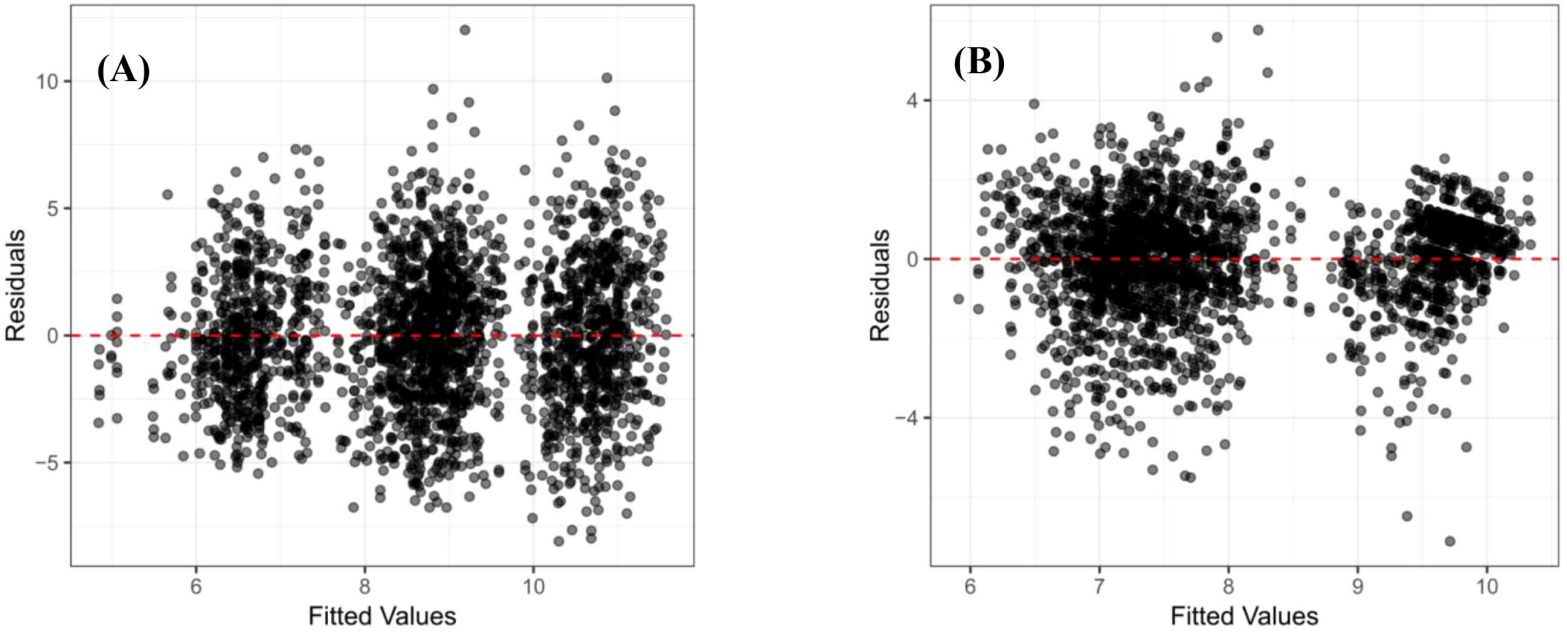
Homoscedasticity test of mixed model residuals: **(A)** is DBH; **(B)** is tree height.

The output of the mixed model operation displayed “REML criterion at convergence: []”, indicating successful model convergence. This was further verified by the message “Convergence achieved (0)”, confirming convergence. The mixed model residuals satisfied the assumptions of normality (**Figure 1**) and homoscedasticity (**Figure 2**). Furthermore, the DBH model demonstrated better performance compared to the tree height model.

**Figure 3:**
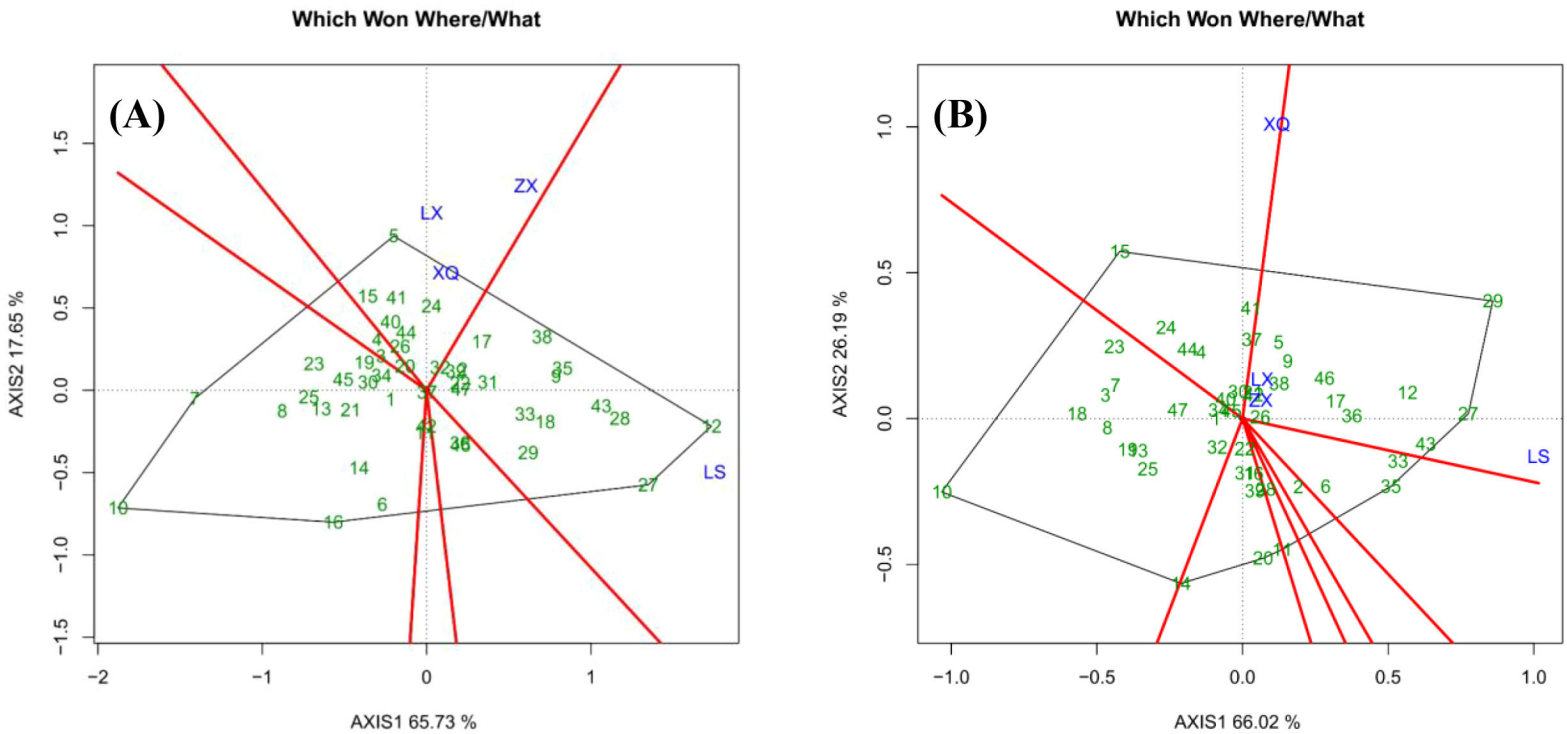
Grouping of the sites and the best family for each site: **(A)** is DBH; **(B)** is tree height.

Significance testing for fixed effects in the mixed model (**Table 3**) revealed that location effects were highly significant (P< 0.001). This indicates strong environmental influences on the 5-year-old DBH and tree height. Regarding random effects (**Table 4**), block effects were non-significant for DBH but highly significant for tree height (P< 0.001). Family and family × location interaction effects were significant or highly significant for both traits (P< 0.05; P< 0.01). These findings demonstrate that DBH and tree height are significantly influenced by genotype and GE interactions.

**Table 3:** Significance testing of fixed factors in mixed linear models.

Trait	Fixed factor	Sum Sq	Mean Sq	NumDF	DenDF	*F*	*P*
DBH	Location	4074.6	1358.2	3	141.95	171.03	<0.001^***^
Tree height		1890.1	630.04	3	145.1	326.09	<0.001^***^

Notes: ***P*<* 0.001

**Table 4:** Significance testing of a random factor in modified mixed linear models.

Trait	Random factor	Npar	LogLik	AIC	LRT	Df	*P*
DBH	Family	7	−6139.0	12292	10.3634	1	<0.01^**^
	Block	7	−6134.7	12284	1.8280	1	0.1764
	Family×location	7	−6136.4	12287	5.1817	1	<0.05^*^
Tree height	Family	7	−4399.1	8812.2	5.580	1	0.0182^*^
	Block	7	−4437.9	8889.7	83.137	1	<0.001^***^
	Family×location	7	−4401.3	8816.6	9.996	1	<0.01^**^

Notes: ***P*<* 0.001; **P*<* 0.01; *P*<* 0.05

Using the R lme4 package, we fitted mixed linear models to assess DBH and tree height across four trial sites, as well as for the combined multi-site data, to estimate breeding values (**Tables 5** and **6**, The complete table can be found in the **Supplementary Materials**). For ranking purposes, multi-site breeding values were employed to evaluate family growth performance comprehensively. We applied a selection threshold of 20% to pinpoint elite families, ultimately identifying the top nine families with superior DBH and tree height.

**Table 5:** BLUP breeding values of DBH in multiple trial sites.

Family	ZX	LX	LS	XQ	Multi-Sites	Rank
38	0.37468	0.45447	0.61224	−0.07096	0.50484	1
41	−0.07385	0.48809	−0.22266	0.70583	0.48305	2
35	0.1695	0.38742	0.80944	0.02611	0.44309	3
5	0.8172	0.36171	−0.50853	0.11824	0.41922	4
43	0.19486	0.2271	1.06897	−0.19191	0.37214	5
9	0.28015	0.08293	0.73025	0.11662	0.34125	6
12	0.84929	−0.52933	1.56457	−0.16253	0.30768	7
27	−0.34534	0.07534	1.54366	0.06222	0.29642	8
24	0.53625	−0.03911	−0.17372	0.33878	0.29616	9

**Table 6:** BLUP breeding values of tree height in multiple trial sites.

Family	ZX	LX	LS	XQ	Multi-Sites	Rank
29	0.02001	0.05592	0.80542	0.50537	0.28418	1
5	0.14684	0.17765	0.07608	0.24283	0.2258	2
43	0.06859	0.058	0.63032	−0.02028	0.21561	3
27	−0.03145	0.06294	0.76895	0.10948	0.17515	4
38	0.07374	0.27126	0.09392	0.09329	0.17108	5
35	0.06084	0.0953	0.52645	−0.18609	0.14517	6
12	0.07805	−0.12402	0.55553	0.17432	0.13293	7
36	0.05381	0.06018	0.36748	0.0449	0.12988	8
41	−0.00459	0.17293	−0.02255	0.36023	0.12584	9

The DBH breeding values ranged from −1.27462 to 0.50484, with 25 families registering values above 0. The top performing families were 38, 41, 35, 5, 43, 9, 12, 27, and 24. For tree height, breeding values ranged from −0.42664 to 0.28418, with 23 families showing values greater than 0. The highest-ranking families were 29, 5, 43, 27, 38, 35, 12, 36, and 41. These selected *Acacia melanoxylon* families demonstrate superior growth traits in both DBH and height, as well as greater genetic potential, making them ideal candidates for large-scale promotion.

### 3.2 Multi-location analysis based on GGE biplot

**Figure 4:**
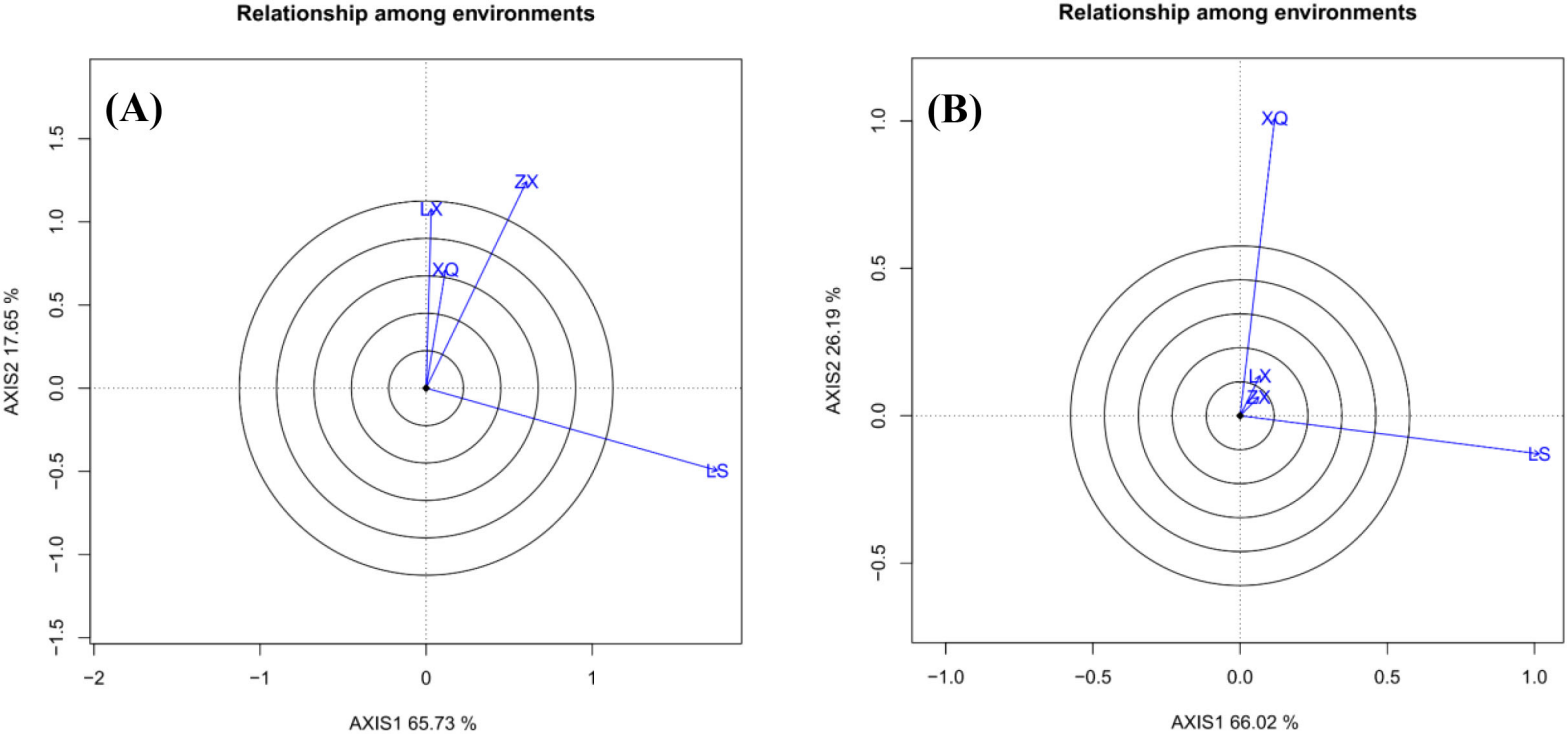
Correlation among the sites: **(A)** is DBH; **(B)** is tree height.

The GGE biplots for DBH and tree height (**Figures 1**-**4**), constructed from the first two principal components (AXIS1 for genetic and AXIS2 for environmental variation), demonstrated high explanatory power, accounting for 83.38% and 92.21% of the total variation, respectively. This indicates that the key patterns of genotype performance and environmental interaction were effectively captured by the model, ensuring the reliability of the subsequent conclusions regarding family selection and site evaluation (Zhao et al., 2022).

#### 3.2.1 Results of site grouping

**Figure 3** presents a “Which Won Where/What” biplot that categorizes trial sites and identifies the most advantageous genotypes within each group. The polygon is formed by the outermost families, with perpendicular lines extending from the origin to partition it into distinct sectors. Each sector’s vertex family represents the top performer for the sites within that sector. In the case of DBH (**Figure 3A**), sites ZX, XQ, and LX constitute Group 1, where Family 5 was the most successful. Site LS forms Group 2, with Families 27 and 12 at the sector vertices; however, Family 12 had the highest BLUP value. Similarly, regarding tree height (**Figure 3B**), sites LX, ZX, and LS make up Group 1, with Family 27 being the top-performing genotype. In Group 2, Site XQ demonstrated Family 15 as the top performer.

We observed a discrepancy between the BLUP rankings and the interpretation derived from the " Which Won Where/What " functional plot. For instance, in the case of DBH, Family 5 was identified as the top-performing genotype in Group 1 based on the vertex of the biplot, whereas Family 38 exhibited the highest multi-site BLUP value. This divergence can be well explained: the multi-site BLUP ranking reflects the comprehensive performance of each family across all four trial sites, while the biplot illustrates their performance within specific environmental groups. Thus, Family 5 demonstrated superior adaptation to the environments in Group 1, whereas Family 38 possessed the highest overall genetic potential.

**Figure 4** “Relationship Among Environments” biplot illustrates the similarity between trial sites during family evaluation. Sites with vectors forming angles smaller than 90° are positively correlated, with smaller angles indicating stronger correlations. Angles greater than 90° indicate negative correlations, while angles around 90° suggest no correlation. In the case of DBH (**Figure 4A**), sites LX and XQ are highly correlated and moderately correlated with ZX, suggesting consistent family rankings. Conversely, site LS is negatively correlated with LX, reflecting opposing rankings. Regarding tree height (**Figure 4B**), sites LX and ZX show a strong correlation, whereas XQ and LS are nearly uncorrelated, with an angle of approximately 90°.

#### 3.2.2 Discrimination and representativeness of the test sites

**Figure 5:**
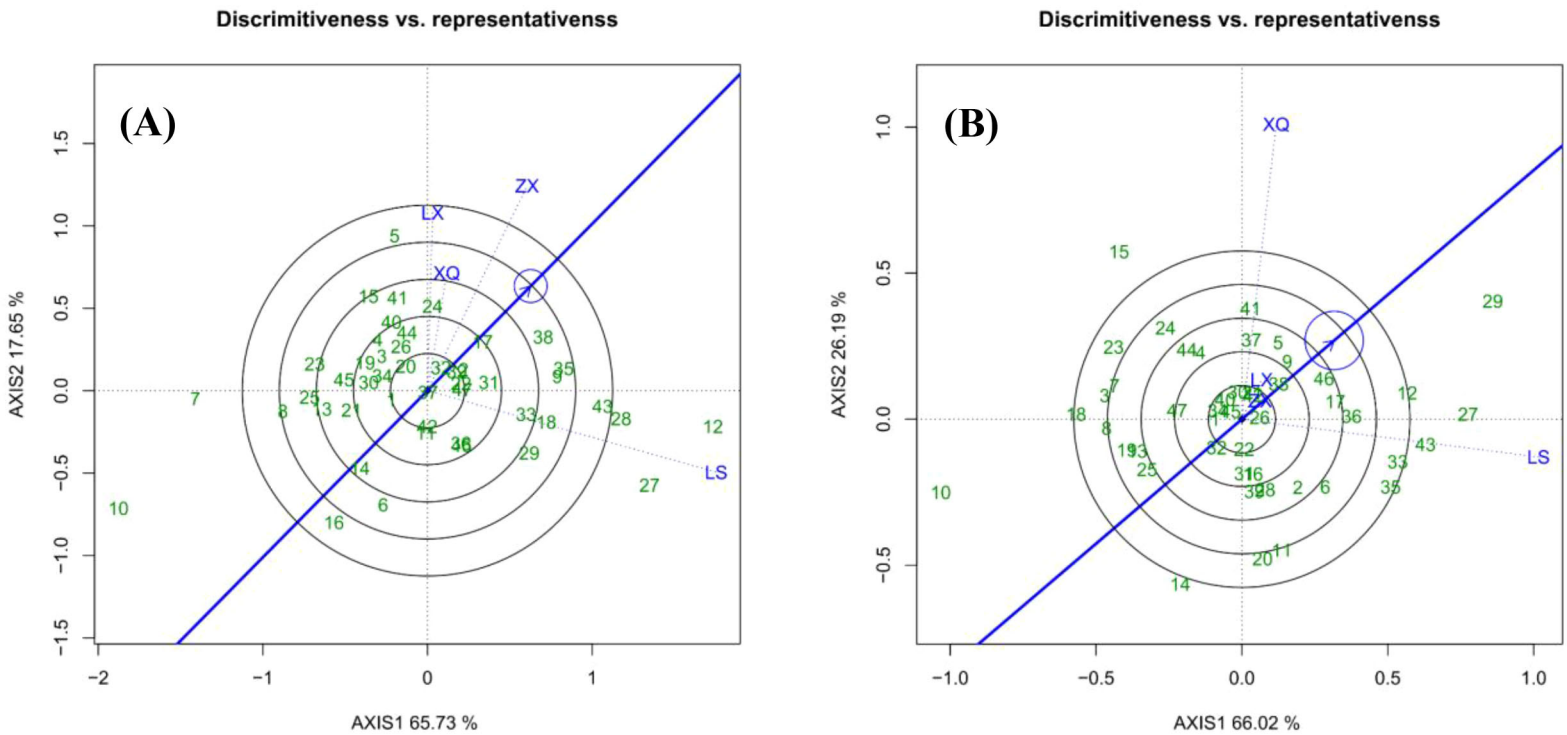
Discrimination and representativeness of the sites: **(A)** is DBH; **(B)** is tree height.

**Figure 5** assesses trial sites in terms of their discriminative power (indicated by vector length) and representativeness (indicated by the angle from the average environment axis). Sites with longer vectors and smaller angles are more effective for selecting high-yield, stable families. In the case of DBH (**Figure 5A**), Site LS exhibited the highest discriminative power, with ZX, LX, and XQ following in sequence. ZX also demonstrated the highest representativeness, followed by XQ, with LS and LX showing similar levels. Consequently, ZX emerged as the most effective site for identifying elite families due to its high representativeness and second-highest discriminativity. For tree height (**Figure 5B**), no trial site could be identified that effectively selects families.

#### 3.2.3 High yield and stability of families

**Figure 6:**
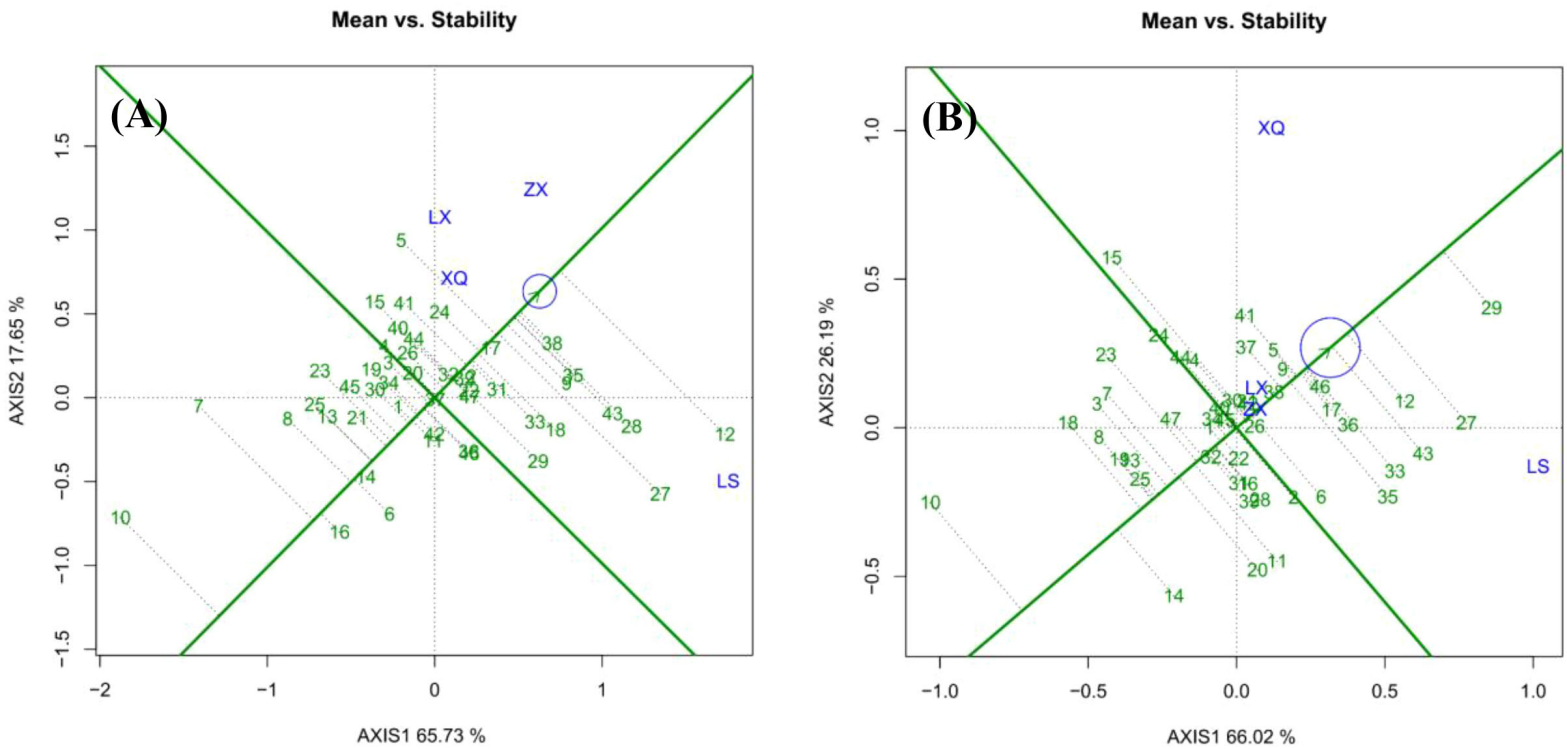
(Mean and stability of the families: **(A)** is DBH; **(B)** is tree height.

Within a specific family ecological zone, ideal families should achieve high yield and stability. The perpendicular line to the average environment axis, which passes through the origin, represents the overall mean growth value of the trait (**Figure 6**). Families positioned on the right side of this axis have growth values exceeding the mean, where a greater distance from the axis signifies a larger DBH or tree height. Conversely, families on the left side display below-average growth, with increased distance indicating smaller trait values.

For DBH (**Figure 6A**), Family 12 exhibited the highest growth, followed by Families 38, 28, and 43. Conversely, Family 10 demonstrated the lowest growth, with Families 7, 16, and 8 also performing poorly. Families 4, 20, and 37 displayed DBH values close to the overall mean. Regarding tree height (**Figure 6B**), Family 29 achieved the highest growth. Again, Family 10 ranked lowest. Families 24, 44, 40, and 2 exhibited tree heights near the overall mean.

The length of the vertical dashed line from each family to the average environment axis indicates stability, with shorter lines signifying greater stability. For DBH (**Figure 6A**), Family 12 exhibited the least stability, followed by Families 27 and 7. Conversely, Family 37 demonstrated the highest stability, followed by Families 17 and 14. Families 38, 35, 9, 33, 31, and 24 showed moderate stability. Concerning tree height (**Figure 6B**), Families 46, 17, 36, 29, 37, and 5 displayed moderate stability. The families within the top 25% growth rate were assessed and chosen for both high and stable yield, with Nos. 38, 35, 9, 17, and 24 demonstrating strong DBH rapidness and stability. Families 46, 36, 17, 5, and 9 were identified as having robust high-speed growth and stability.

## 4 Discussion

### 4.1 Data selection for GGE biplot analysis

Traditional biplots relying on fixed-effects models face limitations in forestry METs due to their assumptions of homogenous environmental variances and balanced data—conditions rarely met in practice given inherent spatial heterogeneity and occasional missing observations (Gauch et al., 2008). Our study substantiates these challenges, as evidenced by the unbalanced number of replicates across our four test sites. To overcome these constraints, we employed a mixed linear model, from which BLUP breeding values were extracted (Ling et al., 2021). The validity of our model was confirmed through convergence checks and residual diagnostics, ensuring that the BLUPs accurately reflected genetic effects (Piepho et al., 2008). The high discriminative power of sites like ZX and the clear differentiation of stable families (e.g., 38, 35) in our resulting biplots empirically demonstrate that BLUP-based data enhance the reliability of stability assessments and selection accuracy compared to analyses based on raw phenotypic data (Ferreira et al., 2012; Amrate et al., 2024).

The GGE biplots for DBH and tree height in this study explained 83.38% and 92.21% of the total variation, respectively, indicating reliable results, with tree height exhibiting higher reliability than DBH. However, considering the breeding objective for large-diameter timber and uncertainties in volume calculation, along with the inability of the "Discriminiveness vs. representativeness" plot for tree height to effectively identify test sites with both high discriminative power and strong representativeness, the GGE biplot analysis for DBH was ultimately adopted as the primary reference. Furthermore, compared to tree height measurement, DBH measurement offers higher accuracy. Genetic correlations among traits suggest that breeders can select high-productivity individuals based on larger DBH, which is easier to measure and more cost-effective (Adams et al., 2007; Kroon et al., 2008; Munhoz et al., 2021) . This further supports the feasibility of prioritizing the DBH-based GGE biplot analysis.

*Acacia melanoxylon* has been introduced to China as an important timber species for promotion. As a fast-growing tree species, its rotation period in China is approximately 8 years. Research on tree trials both domestically and internationally suggests that volume growth can be reliably evaluated at 1/3 to 1/2 of the rotation period. Thus, using DBH data from 5-year-old *Acacia melanoxylon* for evaluation and selection provides valuable reference. Additionally, while this study is based on predictive analysis using 5-year growth data, it should be noted that long-term growth patterns may evolve as trees develop. Continuous monitoring and integration with practical breeding efforts are essential for guiding future promotion and utilization.

### 4.2 Evaluation of tested families and sites

Regional METs are a primary and widely used method in forestry research. By analyzing data from multiple sites, METs facilitate the evaluation of genotype adaptability and stability, the identification of genotypes suited to diverse environments, and the delineation of optimal cultivation zones (Sixto et al., 2014; Nelson et al., 2018). On the average environment axis (**Figure 4A**), Family 12 demonstrated the largest DBH growth but exhibited low stability. In conjunction with breeding value analysis, Family 12 performed optimally at the Luoshan Forest Farm (LS) and moderately well at Zhongxi Forest Farm (ZX), while performing poorly at the other sites. It is recommended for planting in regions with ecological conditions akin to LS and ZX. Notably, stability is only practically significant when it coincides with high yield. For example, although Family 14 showed a “stable” performance, it was categorized as a “stable low-yield” family, making it economically unviable for DBH growth and unsuitable for promotion.

There is a strong correlation among the test sites ZX, LX, and XQ, with the ordering of family performances being similar across these locations, suggesting they may represent a similar mega-environment. This close relationship can be interpreted by examining the environmental data in **Table 1**.

While the soil types (mountain red soil) are similar between ZX, LX, and XQ, and differ from LS’s sandy loam, the climatic data reveal a more compelling pattern. Site XQ, situated in northern Fujian Province, has the lowest annual average temperature (17.3°C) among the four test sites. Notably, sites ZX and LX also experience relatively moderate average temperatures (20.2°C and 19.5°C, respectively) compared to the overall regional variation. Furthermore, the author's on-site investigations confirmed that *Acacia melanoxylon* at these sites grows primarily on shady slopes, which likely maintains a cooler microclimate than the broader forest farm averages might suggest. This consistent exposure to comparatively lower temperatures is the most parsimonious explanation for the strong genetic correlation among sites ZX, LX, and XQ.

In contrast, site LS appears independent and shows a weak correlation with the other three sites. Although LS has a reported average temperature of 20°C, it is located at a substantially higher elevation (415–481 m) compared to ZX (140–250 m) and LX (200–350 m). Higher elevation is often associated with greater temperature fluctuations and cooler conditions, which could differentiate its environmental profile. The combined factors of its distinct soil type and potentially unique microclimate at higher elevation may explain its divergence. However, to definitively pinpoint the causative environmental factors, further controlled experiments and more granular environmental monitoring are necessary.

According to the ranking of multi-site breeding values for DBH, a total of 25 families exhibited breeding values greater than 0. Among them, only 7 out of 17 families from the Australian introduced populations were selected, accounting for 41.18%, while 18 out of 30 families from the Luoxi Forest Farm seed orchard performed excellently, representing 60% of this group. This notable difference highlights the critical role of artificial selection in breeding: the proportion of superior genotypes in the locally pre-selected seed orchard population was significantly higher than that in the directly introduced populations. From the perspective of genetic gain, these results demonstrate that scientific selection can effectively enrich superior genotypes, enhance population growth and adaptive potential, and thereby offer more efficient breeding strategies for forest genetic improvement.

## 5 Conclusion

This study establishes the integrated BLUP-GGE biplot analysis as an effective framework for evaluating genotype-environment interactions and selecting superior genetic material in *Acacia melanoxylon*. The methodology's success in delineating mega-environments and identifying stable, high-performing genotypes demonstrates its substantial potential to streamline breeding programs for perennial trees characterized by long cycles and significant G×E effects.

Beyond its immediate application to *Acacia melanoxylon*, the research provides a validated, efficient strategy for the targeted deployment of genetic resources, which is crucial for enhancing forest productivity and adaptability under changing climatic conditions. Future work should focus on advancing the selected elite material into large-scale breeding programs and extending this analytical framework to assess other critical traits, such as wood properties and stress resilience, as well as to other economically important timber species.

## Data Availability

The raw data supporting the conclusions of this article will be made available by the authors, without undue reservation.
