# Supplementary material for: Genotype by environment interaction for growth traits of families of Acacia melanoxylon based on BLUP and GGE biplot
Source: Front Plant Sci. 2025 Nov 14;16:1656136. doi: 10.3389/fpls.2025.1656136 (PMC12660109; doi:10.3389/fpls.2025.1656136)
Supplement: Supplementary file 1 [file DataSheet1.pdf]

Supplementary Material

1 Supplementary Figures and Tables

1.1 Supplementary Tables

Table 5. BLUP breeding values of DBH in multiple trial sites.

Family	ZX	LX	LS	XQ	Multi-Sites	Rank
1	−0.37344	0.28614	−0.10862	−0.00300	−0.05964	31
2	0.40104	−0.00690	0.11195	−0.27967	0.09641	19
3	0.25203	−0.08160	−0.37679	−0.03689	−0.05321	30
4	0.16220	0.18511	−0.37721	−0.02105	0.04066	24
5	0.81720	0.36171	−0.50853	0.11824	0.41922	4
6	−0.68861	−0.25722	−0.02656	−0.24228	−0.47840	44
7	−0.38719	−0.20058	−1.35176	−0.09275	−0.58613	45
8	−0.40600	−0.05059	−0.78428	−0.09121	−0.40054	42
9	0.28015	0.08293	0.73025	0.11662	0.34125	6
10	−0.83788	−0.54110	−1.64334	−0.73099	−1.27462	47
11	−0.32063	0.09130	0.10875	−0.17672	−0.13072	35
12	0.84929	−0.52933	1.56457	−0.16253	0.30768	7
13	−0.21012	−0.19698	−0.60159	−0.05055	−0.29757	41
14	−0.46770	−0.20799	−0.25282	−0.27618	−0.45904	43
15	0.07467	0.31973	−0.45001	0.56289	0.27447	12

16	−0.61079	−0.61346	−0.35809	−0.34849	−0.80304	46
17	0.40562	0.06215	0.20671	0.11571	0.27622	11
18	0.43570	−0.71378	0.61032	0.24076	0.05792	23
19	−0.09346	0.29281	−0.36953	−0.09821	−0.02504	28
20	0.17754	0.13541	−0.18529	−0.26503	−0.03028	29
21	−0.27227	−0.11465	−0.39756	0.06105	−0.21683	40
22	0.17764	−0.10566	0.15239	0.06714	0.08322	21
23	0.00919	−0.13855	−0.73381	0.10119	−0.18688	38
24	0.53625	−0.03911	−0.17372	0.33878	0.29616	9
25	−0.45386	0.14733	−0.60791	0.03682	−0.19831	39
26	0.10783	0.27216	−0.20916	−0.05237	0.09443	20
27	−0.34534	0.07534	1.54366	0.06222	0.29642	8
28	0.39142	−0.05852	1.12984	−0.25597	0.29090	10
29	−0.07398	−0.33388	0.67920	0.11399	−0.01720	27
30	−0.04336	−0.00895	−0.36042	−0.03580	−0.12557	34
31	0.06446	0.21331	0.37290	−0.05027	0.19734	14
32	0.06573	−0.00156	0.04545	0.27743	0.13908	16
33	0.11027	−0.21968	0.58977	0.17036	0.13545	17
34	−0.10269	0.16046	−0.25645	−0.01180	−0.00850	26
35	0.16950	0.38742	0.80944	0.02611	0.44309	3

36	−0.14515	−0.24957	0.27191	−0.01972	−0.14737	37
37	−0.25449	0.13031	0.07078	0.26296	0.10527	18
38	0.37468	0.45447	0.61224	−0.07096	0.50484	1
39	0.43709	−0.22647	0.04744	−0.08719	0.03181	25
40	0.26999	0.23189	−0.33117	0.02885	0.19079	15
41	−0.07385	0.48809	−0.22266	0.70583	0.48305	2
42	−0.15364	−0.21088	0.05187	0.05293	−0.13340	36
43	0.19486	0.22710	1.06897	−0.19191	0.37214	5
44	−0.07475	0.46611	−0.13006	0.25243	0.27095	13
45	−0.28462	0.25100	−0.44196	−0.02155	−0.08327	32
46	−0.15901	−0.35071	0.27174	0.12461	−0.11630	33
47	0.06850	0.13542	0.20914	−0.16387	0.08305	22

Table 6. BLUP breeding values of Tree height in multiple trial sites.

Family	ZX	LX	LS	XQ	Multi-Sites	Rank
1	−0.08512	0.10006	−0.09220	−0.02001	−0.03078	30
2	0.05668	0.02760	0.21341	−0.21835	0.08464	12
3	0.09036	−0.08624	−0.47855	0.02594	−0.05430	34
4	−0.01751	0.02758	−0.16768	0.20770	0.00645	23
5	0.14684	0.17765	0.07608	0.24283	0.22580	2

6	−0.06660	0.12607	0.30910	−0.21007	0.01811	21
7	−0.05366	0.06858	−0.44821	0.05248	−0.06807	35
8	−0.08505	−0.02965	−0.45214	−0.08126	−0.19680	45
9	0.09046	0.00318	0.12684	0.21060	0.11822	10
10	−0.11172	−0.13319	−0.98682	−0.35816	−0.42664	47
11	−0.03346	0.10199	0.18442	−0.44522	−0.03174	31
12	0.07805	−0.12402	0.55553	0.17432	0.13293	7
13	−0.01258	0.02365	−0.34594	−0.15923	−0.10454	39
14	−0.11074	0.05601	−0.14211	−0.59477	−0.18926	44
15	0.07975	0.14552	−0.49826	0.49523	0.09899	11
16	−0.07824	−0.17550	0.07254	−0.15414	−0.13707	42
17	0.03855	−0.12482	0.31551	0.11601	0.07659	14
18	0.00521	−0.41786	−0.54792	0.00228	−0.22498	46
19	0.01352	0.01727	−0.38282	−0.16098	−0.11009	40
20	0.04409	−0.09994	0.12881	−0.46067	−0.08720	36
21	−0.06976	−0.02128	0.02984	0.10219	−0.03953	33
22	0.01184	−0.12430	0.02528	−0.08583	−0.03709	32
23	−0.05034	−0.04416	−0.46184	0.20054	−0.13484	41
24	0.03087	−0.02767	−0.29748	0.28152	−0.00338	24
25	−0.08395	−0.07501	−0.29573	−0.19939	−0.17914	43

26	0.00783	−0.02298	0.06111	0.01551	0.01998	20
27	−0.03145	0.06294	0.76895	0.10948	0.17515	4
28	−0.01690	−0.15745	0.11618	−0.20975	−0.09263	38
29	0.02001	0.05592	0.80542	0.50537	0.28418	1
30	0.00058	0.05873	−0.02811	0.08208	0.03076	17
31	0.02343	0.12057	0.02197	−0.20552	0.01599	22
32	0.00588	−0.00639	−0.07497	−0.10953	−0.02373	27
33	0.02074	−0.15902	0.55541	−0.05790	0.07361	15
34	−0.03185	0.00388	−0.08434	0.01975	−0.00765	25
35	0.06084	0.09530	0.52645	−0.18609	0.14517	6
36	0.05381	0.06018	0.36748	0.04490	0.12988	8
37	−0.07687	0.06214	0.00198	0.27226	0.02769	19
38	0.07374	0.27126	0.09392	0.09329	0.17108	5
39	0.07609	−0.12545	0.07320	−0.23112	−0.01887	26
40	0.03242	0.01861	−0.06759	0.05424	0.03060	18
41	−0.00459	0.17293	−0.02255	0.36023	0.12584	9
42	−0.07098	−0.05163	0.03469	0.09871	−0.03051	29
43	0.06859	0.05800	0.63032	−0.02028	0.21561	3
44	−0.07209	0.13894	−0.21815	0.19987	−0.02740	28
45	0.02125	0.08136	−0.04677	0.00766	0.05490	16
46	0.03128	−0.07033	0.26618	0.18231	0.08251	13

47	−0.01925	−0.05902	−0.22045	0.01096	−0.08843	37
